# Food Safety and Food Hygiene Knowledge of Hungarian University Students

**DOI:** 10.3390/ijerph21111410

**Published:** 2024-10-25

**Authors:** Viola Keczeli, Melinda Kóró, Vivien Tóth, Tímea Csákvári, Boglárka Bernadett Tisza, Patricia Szántóri, Ágnes Czeglédiné Asztalos, Zsófia Verzár, Andrea Gubicskóné Kisbenedek

**Affiliations:** 1 Doctoral School of Health Sciences, Faculty of Health Sciences, University of Pécs, 7621 Pécs, Hungaryandrea.kisbenedek@etk.pte.hu (A.G.K.); 2 Institute of Nutritional Sciences and Dietetics, Faculty of Health Sciences, University of Pécs, 7621 Pécs, Hungary; 3 Doctoral School of Biology and Sport Biology, Faculty of Sciences, University of Pécs, 7624 Pécs, Hungary; 4 Department of Health Economics and Health Care Management, Institute of Health Insurance, Faculty of Health Sciences, University of Pécs, 8900 Zalaegerszeg, Hungary

**Keywords:** food safety knowledge, food hygiene knowledge, university students, foodborne diseases

## Abstract

(1) Background: Foodborne diseases continue to affect millions of people around the world today, posing a huge challenge to public health. Our aim was to focus on the food safety knowledge and food hygiene knowledge of students at the University of Pécs, Hungary. (2) Methods: A quantitative, online, cross-sectional study was conducted between 15 February 2024 and 10 May 2024. Non-probability, convenience sampling was used. The target group consisted of first- and second-year BSc level degree students of the Faculty of Health Sciences at the University of Pécs (N = 214). The questions of the self-designed questionnaire include sociodemographic data, questions focusing on food safety knowledge, and questions to assess food hygiene knowledge. Statistical analysis was performed using descriptive and mathematical statistical analysis (*p* < 0.05). (3) Results: Participants were aware of foods that pose a food safety risk and were familiar with both the pathogens in food and the groups at risk of food contamination. In terms of knowledge, a higher proportion of second-year students answered correctly (*p* = 0.021; r = 0.657). A significant relationship was found between hygiene habits and age (*p* = 0.035) and place of residence, with most of the students living in dormitories not paying attention to food hygiene (r = 0.094; *p* = 0.046). (4) Conclusions: The level of knowledge of the students was not always satisfactory, so further research and education on this topic is essential to promote safe food consumption.

## 1. Introduction

For as long as humanity has been eating, foodborne diseases have been a problem and a burden on public health. Foodborne diseases cause various medical conditions and occur through food or water consumption contaminated by bacteria and/or their toxins, as well as parasites, viruses, or chemicals. The global prevalence of foodborne diseases is a major public health issue, as both industrial and developing countries are affected. The World Health Organization (WHO) reported [1] that one out of ten people becomes sick due to foodborne diseases globally, while more than 91 million people are affected in developing countries despite various research and intervention measures toward food safety [1,2]. As the WHO reported in 2010, around 600 million cases of foodborne diseases and 420.000 deaths were reported due to the consumption of food contaminated by enteric pathogens worldwide [3]. In the European Union, the most common source of infection was meat and other products containing meat, which were responsible for 5.146 foodborne outbreaks in 2018 [4]. One of the most frequent foodborne diseases in the European Union is salmonellosis, which was also the most recurrent foodborne disease in Hungary, with 3.340 cases in 2022 [5,6]. Another common foodborne disease besides salmonellosis is that caused by the pathogen *Escherichia coli. Escherichia coli* remains a major cause of bacterial infections in humans and animals across Europe, and multidrug-resistant strains are becoming increasingly common [7]. In response to the 2011 *Escherichia coli* outbreak that affected nearly 4.000 people and caused 46 deaths, the European Commission allocated EUR 12 million to enhance research capabilities for addressing such epidemic threats [8,9,10]. In addition to these two pathogens, *Staphylococcus aureus* is one of the most important pyogenic bacteria-causing infections in the community and in hospitals. It can also colonise the skin and mucous membranes of humans and many animal species. According to a 2012 study, about one-third of healthy Hungarian preschool children carry *Staphylococcus aureus* in their noses. This result is in line with findings from other European countries that about 20% of people are persistent and 30% are intermittent carriers of *Staphylococcus aureus* nasal discharge [11].

The available literature has shown that students are looking for independence and to become self-reliant, and with changes in their daily schedules, they tend to pay less attention to food safety awareness and food hygiene [12,13,14]. Previous studies proved that knowledge of food safety and food hygiene rules is crucial for preventing several foodborne diseases [15]. Good practices at home, such as storing food and ingredients at the right temperature, cleaning the kitchen properly, and adequate disinfection and hand-washing techniques, in general, are crucial in fighting infections [16,17]. In Hungary, students usually do not really receive any kind of food hygiene education, so it is crucial to assess their awareness about the topic. Providing them with appropriate educational materials can help them increase their knowledge about food safety. It was assumed that the food hygiene knowledge of students at the University of Pécs is adequate. It was also assumed that this knowledge would increase with the number of years spent as a student, age, or socioeconomic situation (e.g., living in a dormitory, social situation).

The aim of our study is to assess the food safety and food hygiene knowledge of students at the Faculty of Health Sciences at the University of Pécs, and whether they are aware of the rules around safe storage/transportation and heating of risky foods.

## 2. Materials and Methods

### 2.1. Instrument

The quantitative, cross-sectional study was conducted between 15 February 2024 and 10 May 2024. Our target group was the full-time, first- and second-year students of the Nursing and Patient Care Bachelor of Science programme of the Faculty of Health Sciences at the University of Pécs. (The first- and second-year students have not yet taken the microbiology course this semester, so they have not been introduced to this subject at university.) Exclusion criteria included not being an active student, possibly studying at higher years, or working at the MSc degree level. After considering the inclusion and exclusion criteria, a total of 214 participants took part in the survey. Data collection was carried out by means of an online questionnaire, which was sent to students via the Neptun study system newsletter, and face-to-face interviews were also carried out during lessons and breaks. Non-random, convenience sampling was used. The questionnaire was created using the Google questionnaire editor.

### 2.2. Tools

The questionnaire was developed by incorporating questions selected from an updated, reliable, and valid instrument produced by Byrd-Bredbenner et al. [18]. The questionnaire was developed in 2007 to assess food safety knowledge and food hygiene knowledge. The questionnaire consists of 48 questions with multiple choices, divided into two parts. The first part of the questionnaire is structured around questions on general knowledge and theoretical knowledge of food safety, food storage, and vulnerable groups at risk of foodborne diseases. This section on food safety knowledge contains 11 questions with multiple choices. The answers were converted into points. Participants’ answers were scored, and those who answered a question correctly were awarded one point. The scores from the 11 questions were then summed, and the respondents were divided into three groups:

- Low food safety knowledge: ≤6 correct answers out of 11;
- Medium food safety knowledge: 7–9 out of 11;
- High food safety knowledge: ≥10 out of 11.

The second part of the questionnaire includes 14 questions about knowledge of pathogens and safe heating/storage, and whether they knew the rules for handling foods and dishes that pose a risk, such as *“How do you store food for several hours to make it safe for consumption?”*. Based on the results, the outcome of the questionnaire could indicate the following:

- An inadequate knowledge level for those who scored 7 or less out of 14 questions;
- A medium knowledge level for those who scored between 8 and 11;
- A proper knowledge level of focus for those who scored 12 or more.

Questions were asked about knowledge of foods that increase the risk of foodborne diseases and about the groups most at risk of foodborne diseases as well.

The demographic characteristics surveyed include the following:

1. Gender;
2. Age;
3. Residential status;
4. Social status.

Completion of the Hungarian-language questionnaire was completely voluntary and anonymous, and the data were collected and processed in accordance with the GDPR in force in the European Union.

### 2.3. Statistical Analysis

IBM SPSS Statistics 26.0 software (IBM SPSS, Version 26.0, Chicago, IL, USA) was used to process the responses. The demographic characteristics are presented as independent variables (gender, age, residential status, and social status). The results of the food safety knowledge test are considered dependent variables. A 95% confidence interval was defined for the chi-square test and normality test. For non-parametric comparisons, the Kruskal–Wallis tests were used. Furthermore, Pearson’s correlation was used to determine if there is a relationship between knowledge groups and knowledge of food safety rules. The results were considered significant when *p* ≤ 0.05.

### 2.4. Ethics

The research has been approved by the Scientific Vice-Rector of the University of Pécs and by the Institutional Review Board (IRB) (PTE/121225-2/2022). The study conformed to the ethical principles outlined in the Declarations of Helsinki. Before participating in the study, all of the subjects gave written informed consent online.

## 3. Results

### 3.1. Sample Characteristics

This study involved 214 (N = 214) university students, 191 women (n = 191) and 23 male students (n = 23). This male/female ratio is representative of the sex ratio of students studying health sciences. Of those who completed the survey, 117 (n = 117) were studying to become dietitians and 97 (n = 97) were physiotherapist, nursing, and paramedic students. In terms of age, the youngest respondent was 18 and the oldest was 28, with a mean age of 20.35 ± 1.52 years and a median of 20 years. Among the respondents, 40 students (n = 40; 18.4%) live in a dormitory with a private bathroom and kitchen, while 38 (n = 38; 17.5%) live in a dormitory with a shared bathroom and kitchen. The majority, 97 students (n = 97; 44.7%), live in rented accommodation, 32 students (n = 32; 14.7%) live at home with their parents, and 2 students (n = 2; 1%) live in their own home. To further analyse the data in terms of housing, the following groups were created: group 1: living in a dormitory (n = 82; 38.3%), group 2: living in rented accommodation (n = 78; 36.5%) and, group 3: living in their own accommodation (n = 48; 22.4%).

Among the students surveyed, the proportion of first- and second-year students was almost the same (first year: n = 106; 48.1%, second year: n = 111; 51.9%). The majority of students identified themselves as having an average income, accounting for more than 60% of the sample (n = 142; 65.4%). A further 41 students (n = 41; 18.8%) identified themselves as having a below-average income, and 34 students (n = 34; 15.7%) have an above-average income. The majority of students cook 3–4 times a week (n = 121, 55.8%), 28 students cook daily (n = 28;12.9%), and 65 students cook rarely, i.e., every two weeks or every month (n = 65; 30.3%). Most students prepare their own meals (n = 115, 53.7%), 96 (n = 96; 44.2%) students are cooked for by their parents/grandparents or partner, and 2 students (n = 2; 0.93%) regularly use catering services.

### 3.2. Food Safety and Food Hygiene Knowledge

Hygiene knowledge was significantly higher among those aged 25 and over. Also, by sociodemographics, significantly higher food hygiene knowledge was found among those aged 25 and over and those living with family or a roommate. Those living in a dormitory were less likely to pay attention to good practices (Table 1 and Table 2).

Our results show that the higher the students’ level of food safety knowledge, the higher their level of food hygiene knowledge (Pearson’s correlation r = 0.249, *p* < 0.01). Dietitian students are neither more informed nor more attentive to hygiene rules than those with other health-related majors.

There was no significant difference between the results of male and female students in terms of knowledge of the recommended freezing temperature (−18 °C). Regarding the knowledge of the participants, a significantly higher proportion of older respondents and second-year students knew the correct answer regarding freezing temperature. No significant difference was found for any variable in knowledge of the refrigerator temperature (1 to 4 °C). Half of the respondents knew the correct answer. It is important to maintain an optimal temperature in the refrigerator to reduce the growth of bacteria that can cause food poisoning and spoilage.

Also, the internal temperature of heating was correctly answered by half of the students (74 °C). They were also aware of the practices that contribute to food contamination, but despite this, there were several cases where incorrect practices were considered the right way to avoid food contamination. College students were more prone to inappropriate food handling, with higher rates of incorrect responses when shopping for groceries, as well as incorrect responses relating to the correct practice of buying frozen food, how to defrost frozen products, and when to throw them away. It was observed that second-year dietitian students gave more correct answers to these questions (*p* = 0.047).

Students are familiar with the groups at risk of foodborne diseases. They know which foods can cause disease in a given group. In terms of knowledge, a higher proportion of second-year students answered correctly (*p* = 0.021, r = 0.657). More than 46.7% of the respondents said they paid more attention to hygiene rules when buying, labelling, and processing food. The highest proportion of correct answers to these questions was given by second-year dietitian students (*p* = 0.039). The most known foodborne pathogens among the students were *Salmonella typhi*, *Staphylococcus aureus*, and *Escherichia coli.* In addition to these, dietetic students also knew *Listeria monocytogenes* and *Clostridium botulinum* as foodborne pathogens, while students from other disciplines knew none or very few (4.6%). The lowest number of correct answers was for the correct knowledge of kitchen hygiene rules, with no differences found for only one variable and with a higher proportion of correct answers given by second-year dietetic students (*p* = 0.041).

## 4. Discussion

The number of foodborne diseases in the European Union, including Hungary, is accurately documented and still high [1]. University students represent one of the most important groups in society, as they will certainly be the graduates and intellectuals of the future. In addition, society should not forget the fact that they want to be individuals and independent from their families, so they create their own hygiene rules themselves. However, as young adults, these future rules can be shaped by education, which is why students were chosen as the target group for this study. Of the respondents, 46.7% paid a lot of attention to hygiene rules when shopping, keeping food at the right temperature, and processing food. Housing conditions have a significant impact on knowledge and adherence to food hygiene rules. As previously hypothesised, students living in dormitories are less attentive to good practice.

A limitation of this study is that 117 (n = 117; 54,8%) respondents of the 214 students who completed the questionnaire are dietetic students, and 51.2% (n = 97) are sophomore students who are more likely to be more experienced in hygienic rules based on their courses than students from other majors. A survey of university students in Spain also shows that students studying health sciences, particularly nutrition and dietetic undergraduates, have the highest knowledge of food safety. Students in other areas of health science are in second place [19].

A 2011 study among university students in Greece and a 2010 study among female university students in Jordan showed that students in health sciences, including those in higher years of their studies, acquire knowledge about food safety and good food handling during their studies [20,21]. For this reason, their knowledge is more relevant than that of those who are not studying health sciences and those who are freshmen in the faculty. Similarly, a joint survey by Novi Sad University and Belgrade University showed higher knowledge among senior students [12]. However, there was no difference between students’ food safety attitudes and the number of semesters they had completed at university in a survey conducted by Trakia University in Bulgaria [22]. Studies on similar topics help to highlight that basic knowledge, such as basic food hygiene, which is thought to be essential and which is also linked to everyday activities, is often still lacking in young people today. A possible way to extend our research is to include students from abroad or from other faculties of our university in the survey. Students form different hygiene habits based on their different culture backgrounds and may have different knowledge on the subject, which may vary considerably on an international level as well.

## 5. Conclusions

Our research aimed to assess students’ food safety and food hygiene knowledge. The types, severity, and impact of diseases have changed over time, and they show multicultural diversity not only by country but also by region and community. An important pillar of prevention is to assess the level of knowledge of all sections of society. The present study focused on university students, with the aim of using the findings to develop targeted educational materials, especially for students with low knowledge levels, to reduce the incidence of foodborne diseases.

Our results are of great importance for changing poor food safety knowledge and poor food hygiene knowledge related to food habits and are therefore important for health promotion and the prevention of underlying diseases.

## Figures and Tables

**Table 1:** Sociodemographic data versus food safety knowledge (N = 214).

Variable	Total N	Low Safety Knowledge N (%)	Middle Safety Knowledge N (%)	Satisfactory Safety Knowledge N (%)	*p*-Value
**Gender**					
Female	191	24 (12.56%)	96 (50.31%)	71 (37.11%)	0.237
Male	23	3 (13.04%)	10 (43.51%)	10 (43.51%)	
**Age groups (years)**					
18–20	137	14 (10.22%)	75 (54.74%)	48 (35.04%)	0.041 *
21–24	71	9 (12.68%)	33 (46.48%)	29 (40.85%)	
25<	6	1 (16.67%)	1 (16.67%)	4 (66.67%)	
**Income status**					
Average	143	17 (11.89%)	70 (48.95%)	56 (39.16%)	0.084
Below average	36	3 (8.33%)	20 (55.56%)	13 (36.11%)	
Above average	35	4 (11.43%)	19 (54.29%)	12 (34.29%)	
**Grade**					
1st year	103	12 (11.65%)	57 (55.34%)	34 (33.01%)	0.046 *
2nd year	111	12 (10.81%)	52 (46.85%)	47 (42.34%)	
**Major**					
Dietitian	117	17 (14.53%)	57 (48.72%)	43 (36.75%)	0.251
Other health specialisations	97	7 (7.22%)	52 (53.61%)	38 (39.18%)	
**Place of residence**					
Dormitory	88	11 (12.51%)	42 (47.73%)	35 (39.77%)	0.064
Own apartment	48	3 (6.25%)	26 (54.17%)	19 (39.58%)	
Rented apartment	78	10 (12.82%)	41 (52.56%)	27 (34.62%)	

* Significant at 0.05.

**Table 2:** Sociodemographic data versus food hygiene knowledge (N = 214).

Variable	Total N	Low Hygienic Knowledge N (%)	Middle Hygienic Knowledge N (%)	Satisfactory Hygienic Knowledge N (%)	*p*-Value
**Gender**					
Female	191	33 (17.28%)	138 (72.25%)	20 (10.47%)	0.619
Male	23	3 (13.04%)	18 (78.26%)	2 (8.70%)	
**Age groups (years)**					
18–20	137	23 (16.79%)	102 (74.45%)	12 (8.76%)	0.035 *
21–24	71	13 (18.31%)	50 (70.42%)	8 (11.27%)	
25<	6	0 (0%)	4 (66.67%)	2 (33.33%)	
**Income status**					
Average	143	29 (20.28%)	100 (69.93%)	14 (9.79%)	0.379
Below average	36	3 (8.33%)	30 (83.33%)	3 (8.33%)	
Above average	35	4 (11.43%)	26 (74.29%)	5 (14.29%)	
**Grade**					
1st year	103	17 (16.50%)	77 (74.76%)	9 (8.74%)	0.643
2nd year	111	19 (17.12%)	79 (71.17%)	13 (11.71%)	
**Major**					
Dietitian	117	23 (19.66%)	82 (70.094%)	12 (10.26%)	0.052
Other health specialisations	97	13 (13.40%)	74 (76.29%)	10 (10.31%)	
**Place of residence**					
Dormitory	88	16 (18.18%)	64 (72.73%)	8 (9.09%)	0.012 *
Own apartment	48	8 (16.67%)	35 (72.92%)	5 (10.42%)	
Rented apartment	78	12 (15.38%)	57 (73.08%)	9 (11.54%)	

* Significant at 0.05.

## Data Availability

The datasets will be made available upon reasonable request.
